# Parents’ and Early Childhood Educators’ Perceptions on Movement and Learning Program Implementation

**DOI:** 10.3390/ijerph182211913

**Published:** 2021-11-13

**Authors:** Myrto F. Mavilidi, Sue Bennett, Fred Paas, Anthony D. Okely, Spyridoula Vazou

**Affiliations:** 1 School of Education, University of Wollongong, Wollongong, NSW 2522, Australia; sbennett@uow.edu.au (S.B.); paas@essb.eur.nl (F.P.); 2 Early Start, University of Wollongong, Wollongong, NSW 2522, Australia; tokely@uow.edu.au; 3 Department of Psychology, Education & Child Studies, Erasmus University Rotterdam, 3025 Rotterdam, The Netherlands; 4 School of Health & Society, University of Wollongong, Wollongong, NSW 2522, Australia; 5 Department of Kinesiology, Iowa State University, Ames, IA 50011, USA; svazou@iastate.edu

**Keywords:** video-based programs, delivery, physical activity, preschool children

## Abstract

There is currently limited evidence on parents’ and early childhood educators’ perspectives on implementing programs that combine cognitive and motor tasks in early childhood. An online survey was distributed across Australia through social network platforms and emails at preschool centres, asking 65 parents of preschool children and early childhood educators about their preferences on program delivery, duration, and mode. Responses from the survey were evaluated in order to develop and pilot a 4 week home-based (*n* = 5 parents) and a 6 week school-based program (*n* = 5 educators) including cognitively engaging physical activity, requesting parents’ and educators’ perspectives, respectively, about the program components. Results from the online survey showed a preference for programs with online (e.g., video-based) compared to traditional delivery (e.g., books), emphasising the potential benefits on children’s physical activity levels, sleep, and cognitive function. However, after piloting the program, educators preferred to use the book version instead of the video. This program has the potential to become part of daily regular practice. Barriers reported include logistics issues (i.e., book size), connectivity issues with internet, and the need for varying activities.

## 1. Introduction

The preschool years (under 5 years) are a critical time for cognitive and behavioural growth, as well as for dynamic and elaborative developmental changes in children’s brain [1]. This period is also important for promoting a healthy lifestyle, including physical activity participation [2]. A systematic review of research in children under 5 years showed that physical activity was associated with improved motor development, bone and skeletal health, and fitness [2]. Preschool children also showed improved cognition and psychosocial health as a result of physical activity, with greater evidence seen for children aged 3–4 years. 

The World Health Organisation’s [3] physical activity guidelines for children in preschool years (3–5 years) recommend a minimum of 180 min of physical activity during the day, of which at least 60 min should be spent in moderate- to vigorous-intensity physical activity. Preschools and schools are considered ideal settings for promoting physical activity, as they are equipped with the necessary resources and staff [4].

However, lack of educators’ time during the school day, limited opportunities for training and professional learning development, limited accessibility of children and preschools to natural settings, as well as safety concerns were reported as barriers by early childhood educators for providing limited opportunities for physical activity participation in preschool services [5,6,7,8]. Other barriers that have been reported include the lack of non-standardised curriculum and context-specific physical education teacher education in early childhood programs, and lack of educator’s confidence [9]. 

Early childhood educators’ own perceptions (e.g., lack of interest) and personal negative experiences with physical activity can also explain why they can be reluctant in promoting movement sessions during the preschool day [9]. If we want to engage and convince early childhood educators to implement physical activity programs in preschools, it is important to raise their awareness of the benefits of physical activity in young children during their stay at the preschool, which can lay the foundation for a healthy lifestyle [6]. Early childhood educators suggested that ways to increase physical activity participation should include more field trips, educator knowledge of the benefits and delivery of classroom-based physical activity, implementation of movement breaks, physically active lessons, movement transitions between activities, and modelling motor vocabulary (e.g., asking motor-related questions, include prompts related to motor actions [6,8,9,10]. 

Creating a supporting environment which adopts whole-of-school physical activity policies could be helpful for children’s health and development [8]. Successful strategies to generate positive changes in the educational context and promote healthier lifestyles in children involved better arrangement of the physical environment and actively involving members of the school community and parents [11,12]. In addition, increasing educators’ personal intrinsic motivation and feelings of professional responsibility were also found to be effective [11,12]. Importantly, because parents’ practices and habits are the main determinants for development and establishment of obesity-related behaviours in children 0–5 years, suggesting ways to adopt healthy behaviours is imperative [13]. 

Several systematic reviews and meta-analyses focusing on active learning (e.g., active lessons, active breaks, or cognitively engaging physical activity) found that it can improve learning and increase physical activity levels in preschool and primary school children [14,15,16,17]. For example, preschool children’s memory performance and physical activity were enhanced by integrating physical activity in learning tasks (e.g., foreign language, mathematics, geography, and science [18,19,20,21]. Two narrative reviews have also shown that programs combining motor and cognitive tasks, may offer physical and cognitive benefits, such as improved executive function skills and academic achievement, compared to programs involving only physical activity [22,23]. Executive functions, defined as cognitive control functions necessary for concentrating and thinking, need explicit training. The core executive functions include inhibition (i.e., ability to stay focused and resist temptations), working memory (i.e., ability to hold information while working on it), and cognitive flexibility (i.e., the ability to switch quickly focus of attention [24]). For instance, Schmidt et al. (2020) [25] trained preschool children’s executive functions while they performed games with gross motor movements (e.g., “Lizard Ezi says” to jump up and turn quickly in circles). Children in the movement condition showed greater improvements on their updating performance, compared to children in a control group who continued the usual practice. 

Notably, the preschool environment is unique as it is the first structured environment most children engage in and it is critical for developing positive attitudes towards school and learning, as well as following rules [26]. Active learning programs in the preschool classroom have the potential to provide rich learning experiences but their success depends to a large extend on their degree of implementation. If teachers perceive more barriers than facilitators in implementing active learning programs, they are more likely not to implement those programs [27]. Therefore, the path to implementation could benefit from research that seeks to uncover solutions to existing barriers for active learning programs [28]. 

Emerging evidence on early childhood educators’ perceptions is quite recent, exploring merely opportunities for physical activity practices in general, whereas evidence for parents’ perceptions is scarce. However, the role of parents is important to be considered as they are making final decisions about their children and have an ongoing interaction with educators. Although there is a growing number of active learning programs, there is disperse knowledge on the facilitators and barriers in applying those programs in the preschool environment, with the main focus in the literature being on barriers and facilitators in the elementary classroom [10,27,29]. As such, this study focused on perceptions of early childhood educators and parents of preschool children on programs combining motor and cognitive tasks. In addition, we were interested in their specific feedback on a pilot program we tested. We aimed to explore possible barriers and facilitators in order to understand and respond to educators’ and parents’ needs.

## 2. Materials and Methods

### 2.1. Study Design

A two-phase approach was used in this study, with results from Phase 1 informing Phase 2. In Phase 1, an online anonymous survey was developed targeting early childhood educators’ and parents of preschool children from the community throughout Australia (across all states). The questionnaire requested participants’ demographic information as well as their thoughts on implementing programs that combine motor and cognitive tasks in early childhood, including early childhood and care (ECEC) services as well as at home. In Phase 2, we designed an intervention with cognitively engaging physical activity. We conducted a pilot study of a 4 week home-based program with parents. In addition, we trialled the same program as a 6 week intervention in ECEC services. Parents and educators provided feedback on the programs accordingly responding to a questionnaire sent to them via email.

### 2.2. Participants

Phase 1 participants included parents of preschool children and early childhood educators from the general community located in Australia including both urban and rural areas. Ethics approval from the University’s Human Research Ethics Committee was sought and received for both phases (Ethics No Phase 1: 2020/332; Phase 2: 2020/261). A participant information sheet, containing a consent form, was attached at the beginning of the online questionnaire for Phase 1 participants. Participants were requested to (digitally) complete and return the consent form, before being able to proceed with the questionnaire. In Phase 2, a participant information sheet, containing a consent form, was given in hard copy to parents and early childhood educators of participating ECEC services. Parents for the home-based program were recruited from the memberships or casual visits of the Early Start Discovery Space of the University of Wollongong, whereas early childhood educators were recruited from invitations sent to directors of local ECEC services. Participants were asked to sign and return the consent forms within a 2 week timeframe from the beginning of this study. Phase 2 participants (home-based and preschool programs) received a book related to the activities, as compensation for their participation. 

### 2.3. Procedure

#### 2.3.1. Phase 1—Online Survey

The survey materials were created using the REDCap (version 10.6.5; 2021 Vanderbilt University) platform, were anonymous and had a total duration of 5 min. The link to complete the survey was circulated via social media communication (i.e., Facebook Early Childhood Educators and Early Start community private groups), emails to directors of the preschool centres across all states in Australia, advertised in the official webpage, as well as in Newsletters and Engagement and Network Centres of the University of Wollongong, Australia. 

It consisted of 18 questions in total: 9 questions with multiple choice options regarding participant demographic information (e.g., gender, age, education and location of residence or work), and 9 questions with pre-filled answers (when needed there was also an option for open-ended responses) about perceptions on the potential of applying programs involving motor and cognitive tasks in early childhood. The survey was slightly altered in order to provide questions relevant to both groups (parents and early childhood educators). Example questions included “Would you prefer the program to be delivered in a hard copy or electronic version?” and “Do you consider programs that combine physical activity with learning tasks as an effective way to reduce sitting time in young children?”. The survey questions are provided in Appendix A.

#### 2.3.2. Phase 2A— Program Development

A program with cognitively engaging physical activity for preschool children was developed, targeting to enhance their executive function skills and physical activity levels, in alignment with the Early Years Learning Framework [30]. The Early Years Learning Framework is the guiding curriculum document of educators in Australia for children from birth until 5 years, attending early childhood and care services (ECEC [30]). The current program incorporated three out of five key outcomes from the Early Years Learning Framework aiming to support children’s learning including children having “a strong sense of wellbeing” (i.e., social, emotional, health and physical wellbeing), are “confident and involved learners” (e.g., “learning through play”, experiential learning), and are “effective communicators” (i.e., emphasising the need for digital literacy and use of digital technology). 

This program involved videos with cognitively engaging physical activity for preschool children. Its design considered that movement-based experiences adjusted in digital environments can provoke children’s exploration and imagination, and in turn instigate their cognitive development [31]. It was also important to combine high levels of relevance and integration between the movements and cognitive tasks (i.e., motor and cognitive tasks are related in terms of content and occurred simultaneously) to elicit paramount learning gains [22].

The program consisted of two versions: a 4 week home-based program for parents and a 6 week school-based program for early childhood educators. After the completion of the 4 week home-based program, the 6 week school-based program begun. The program components were similar for both versions. The same videos were shown at ECEC services as in the home-based program. Considering the feedback from the home-based program, the school-based program did not change design or content. However, it included more activities, and the option of using the video, the book, or both. 

Parents and early childhood educators were asked to play the video and participate in the activities with their children twice per week (at a time convenient to them). The videos included reading sessions and self-explaining activities based on the book “Quincey Quokkas Quo”. This book has been previously shown to support preschool children’s executive function skills [32]. The book embedded activities to train executive function and basic numeracy skills, presented within a story, with set obstacles that children had to overcome to help the main character reach its final goals. At the end of each page/activity, an additional counting activity was involved (e.g., let’s count how many frogs you can see in this page). After 2 and 3 weeks for the home and school-based programs, respectively, variations for progressively more complex activities were included. The videos lasted approximately 15 min. Table 1 shows the timeline per phase and Table 2 portrays the program components with examples of activities for different intervention groups.

#### 2.3.3. Phase 2B— Post-Program Evaluation Feedback

Parents and early childhood educators were not aware of the group assignment and the existence of different groups. At the end of the program, they were sent an email and asked to provide their feedback on the program, responding on 9 open-ended questions (e.g., “What is your overall experience? Did you/your children enjoy the program?”). Similarly, the same questionnaire, including some variation to address the different context, was distributed to early childhood educators via email, after the end of the implementation of the 6 week program. This was adapted from a previous post-program evaluation questionnaire targeting primary school teachers’ perceptions on a program that integrated physical activity within English lessons [33]. The questions of the evaluation feedback are provided in Appendix B.

### 2.4. Statistical Analyses

Participant answers were subjected to thematic analyses in order to identify themes and patterns, describe and interpret their meaning [34]. A deductive approach, using previous research (facilitators and barriers [11]), the COM-B system (capability, opportunity, motivation to understand behaviour [35]), and the movement integration typology (design, strategies, support, and delivery [36]), along with inductive techniques, were used to analyse the data. Common answers were coded and then subsequently organised into higher and lower order themes by the first author. The last author checked the coding and changes were made to come to an agreement between the two authors as needed (MM and SV). Verbatim quotations were taken directly from participants’ answers. 

## 3. Results

### 3.1. Overall Thematic Analysis

Appendix C presents a summary of the thematic analysis and the quotes from both phases. Unique benefits emerged on all forms of design of the program (teacher/parent-driven, research-teacher collaboration, or research/video driven). The program inspired parents to expand on integrated activities in daily reading with their children (parent-driven), whereas the research/driven approach gave freedom for independent implementation as well as for shy children to avoid interactions with “strangers”. A design that would support a research-teacher/parent collaboration was also found beneficial as it facilitates better communication with the research team, making adjustments on explaining, modelling, and delivering the activities. 

Combining motor and cognitive tasks offered innovative ways and opportunities for both parents and early childhood educators to engage with children compared to traditional story time (e.g., adding challenges that kept the interest and engagement continuing). The need for support was emphasised in several quotes by both parents and early childhood educators regarding resources (e.g., iPad use and physical copy of the book, songs, hands-on activities, extra videos), additional ideas by the research team, extra training (e.g., “…so that we are better equipped to implement the program”). 

Although the delivery dose (i.e., twice per week) and time commitment were appropriate, there was a clear preference towards the book over the videos. This was enhanced by technical difficulties that both parents and early childhood educators faced regarding the small size of the book (dimensions: length = 24.2 cm, height = 24 cm, and width = 0.4 cm; weight = 6.4 ounces) during the video, along with the background pictures which were considered as distractions. 

Motivation was another theme that came across from the analysis. Several positive sub-themes were identified as motivational factors for the children and the teachers from the implementation of the program, including increased enjoyment, confidence, and interest, whereas negative factors were not absent, including lack of variety, differences in the student learning style, repetition, task difficulty and class programming. Fewer parents and early childhood educators did not notice any changes on motivational factors. In addition, the academic, cognitive, and motor benefits from the program were emphasised by several teachers regarding improvements on counting, reading, comprehension, reaction time, holistic development, motor skill, and alignment with the Early Years Learning Framework [30]. 

On the other hand, child attendance at preschool (or lack of attendance due to illnesses), time management issues due to staff rotation, and the use of accelerometers as an additional measurement tool, were considered as implementation barriers by early childhood educators. Lastly, it was suggested that the program could be useful as an opportunity for connections with parents and childcare centres, as reported by parents and early childhood teachers. 

### 3.2. Analysis per Phase

#### 3.2.1. Phase 1. Online Survey

Phase 1 included 65 participants. Four participants did not specify if they were parents or early childhood educators and were excluded from the analysis. Demographic characteristics for Phase 1 participants are displayed in Table 3. Participants’ responses were organised based on three thematic units related to (1) program delivery, duration, and frequency, (2) benefits, and (3) barriers.

- Program delivery, duration, and frequency: When participants were asked about their preferences on program delivery, 26 early childhood educators said that they would prefer an electronic form and 18 a hard copy version of programs. Similarly, 10 parents preferred an electronic form and 7 a hard copy version of programs.
- Program duration (see Table 4): Most early childhood educators and parents stated that the ideal duration of a program was 15–30 min (*n* = 14 educators; *n* = 6 parents), or 10–15 min (*n* = 11 educators; *n* = 5 parents). Regarding request on recording and reporting children’s daily behaviours to research staff (sleep time, screen time, physical activity, engagement and enjoyment during the program) for 4–6 weeks, few early childhood educators stated that they were able to provide this information daily (*n* = 3), while most preferred 2–3 times a week (*n* = 8), weekly (*n* = 17), or once a fortnight (*n* = 9). Few parents also stated that they were able to provide the following information daily (*n* = 2), while most of them preferred 2–3 times a week (*n* = 5), weekly (*n* = 6) delivery. There were no parents preferring a fortnight update. Lastly, early childhood educators preferred to be contacted by research staff through email (*n* = 29), text message on their phone (*n* = 7), group chat (*n* = 1), mobile app (*n* = 2), social media (*n* = 3), logbook diary (*n* = 1). Parents stated that the best way to be in contact with research staff is via email (*n* = 6), text message on their phone (*n* = 5), group chat (*n* = 3), mobile app (*n* = 3), social media (*n* = 2), logbook diary (*n* = 1).

- Benefits: When participants were asked if a video-based program showing physical activities combined with learning tasks during screen time would be considered as an engaging way to spend quality time with children instead of traditional videos or sedentary passive video games, a positive response (i.e., yes) was given by 60% of early childhood educators (*n* = 26), whereas 15.9% (*n* = 7) responded negatively. Additionally, 64.7% of parents responded positively (*n* = 11; 7 were missing).
  Two early childhood educators and one parent commented on the potential for more quality time with children (e.g., “Involving physical activities in video-placed programs would be more beneficial than video games.”). In contrast, five early childhood educators reported that they do not use screen time as it is not considered an engaging way for all children (e.g., “I don’t like to use screens around the children in our centre. They have enough of that out of hours.”).
  In terms of potential benefits arising from combining physical activities with learning tasks (see Table 5), early childhood educators and parents reported better physical activity outcomes, cognitive function and sleep. Most early childhood educators believed that combining motor and cognitive tasks was a way to reduce sedentary behaviours (*n* = 33) and improve self-regulation (*n* = 31) in young children. Similar approaches were expressed by parents for reducing sedentary behaviour (*n* = 11) and improving self-regulation (*n* = 9).

- Barriers: Implementation barriers on physical activities combined with learning tasks reported by early childhood educators were related to time restrictions (*n* = 12), space restrictions (*n* = 9), special equipment required (*n* = 9), need for training (*n* = 9). Few early childhood educators (*n* = 12) reported no barriers. Similarly, five parents reported time restrictions, four space restrictions, five the need for special equipment, and one parent the need for training. Additionally, one parent reported no barriers, four parents commented having children with disability, weather conditions (“high temperatures during summer for outdoor activities”) and lack of awareness of the importance of physical activity, as extra barriers (“I feel the childcare centre my child attended lack enough programming, particularly in this area”).

#### 3.2.2. Phase 2. Post-Program Evaluation Feedback

Participants included 5 parents from the 4 week home-based program and 5 early childhood educators from the 6 week school-based program. One ECEC service had 2 educators working on different days of the week (they responded on the questionnaire together). Quotes reported by parents and early childhood educators from the cognitively engaging physical activity group were indicated as cognitively engaging physical activity (CPA), whereas those from the cognition group as COG.

- Program experience, benefits and enablers: The program was considered a positive experience for all parents and educators (*n* = 10). Parent 1 (CPA): “It was a great experience and inspired me to use similar activities in daily reading/play to help my child to develop his math and other cognitive ability.” Parent 1 (COG): “The time commitment was reasonable, the activities were enjoyable and the length of the videos and expectations around record keeping was achievable.” Educator 1 (COG): “The research project was a way to validate learning we see every day”. Specific program elements that parents and educators appreciated included the flexibility of the program, its components and duration of activities, and the engagement with the research team. Parent 1 (COG): “the video was a simple delivery method allowing us all to engage with the activity. Having the flexibility to watch the video at any time was a distinct pro for us. The video was also an appropriate length, it didn’t drag on and we could all maintain our attention. The video narrator came across warm and friendly, encouraging our kids to participate.” Educator 1 (COG): The book itself was interesting and interactive to older children in the group”. Educator 1 (CPA): “Engaging in professional discussions with the team of researchers. The opportunity to contribute to building a local research base of knowledge.”
  Parents (*n* = 3) and educators (*n* = 2) in the cognitively engaging physical activity group liked the combination of physical and cognitive tasks. Parent 2 (CPA): “I like how it embeds math in activities and makes it interesting to children. I feel my son masters the concept better with physical activities and it also trains his reaction (like doing the opposite movement, remembering the order, etc.)”. Educator 2 (CPA): “We loved the movements with the book and the children were able to pick up the movements quickly when we were reading. We love that literacy and physical movement can increase children’s movement skills and seen this through the program”.
- Program implementation issues: The main conclusion by parents and educators was to improve the size of the book during the video as it was not ideal. Educator 2 (CPA): “Just for the video to be clearer… we found it easier to read the children the book ourselves and then do the movement instruction as we read. The video story was hard to hear for a larger group of children”. Two parents also mentioned that a variation of more activities and videos would keep children more engaged. Parent 2 (CPA): “By using a different story each time as my son lost his interest after he watched the same story 3 times.”
  Program barriers were linked to keeping children motivated and engaged (*n* = 2), adherence to dosage and compliance (*n* = 2). Educator 2 (CPA): “A barrier was definitely motivation of the children to engage in repetition using the same book, which then challenged my motivation!” Educator 1(COG): “Having to do the same thing each Tuesday/Wednesday. Our program is very flexible and our groups tend to be from the children’s interests or a happening during the day. Needed the same resource for approximately 3 or 4 small groups. Also, ensure every educator is delivering the program as intended”.
- Program impact: Most parents and educators found an improvement on their children’s counting skills (*n* = 6). Parent 3 (CPA): “He did enjoy it more with repetition, once he understood what was expected. He became better at the games, was predicting what would come next, and his counting also improved a little bit.” Educator 2 (CPA): “We have seen an improvement in our children’s interest and enthusiasm as well as improved counting throughout the program and still now. The children are also asking for the book to be read so they can do the movements”. Educator 1 (COG): “No noticeable difference, maybe some in number work”.
- Program enjoyment: The majority of children seem to have enjoyed the program as reported by parents and educators (*n* = 6). Parent 1(CPA): “He enjoyed it while he was doing it, I just had to motivate him to start sometimes.” Parent 3(CPA): “He did –enjoy—the activities more than the story (which was a bit hard for him to follow on the video—especially with English not being his first language).” Educator 2 (CPA): “Most of the children enjoyed the program as it was something new and they liked the movement”. Educator 2 (CPA): “We have seen children engaging in these experiences with enthusiasm and we have seen children having more interest in numbers and counting”. Educator 1(COG): “The children did seem to enjoy the program. The tasks were challenging and could be varied”. They were also some mixed feelings on children’s enjoyment. Educators 2–3 (COG): “Only some of the children enjoyed the program.”
- Program adoption: Most parents and educators were positive in continuing the activities at home and at school beyond the score of the research project. Parent 1 (CPA): “Possibly, as it would encourage me to do more reading at active times of day, rather than as a calming activity.” Parent 2 (CPA): “Yes, it provided many good examples about how to combine the activity, math, and reading together. I will try to use the similar approach/activities when I read books to him.” Parent 3 (CPA): “Yes. I like that the videos give different options using the same book, but I do prefer doing them while reading the book. The activities are fun and useful, and my 3 kids are enjoying doing them together.” Educator 1 (CPA): “It is something already embedded in our practice, physical activity supports learning retention, allows for modulation of children’s arousal levels supporting regulation. As such, it could potentially be applied as a daily practice in preschools”.
  However, three educators were more sceptical. Educator 2 (CPA): “It would also be a useful tool to share with families to transfer this learning to home. I think this book is best used in a one on one or small group which would enable the adult to support the child where they were currently at”. Educator 1 (COG): “I think it has potential to be part of the program. We see the value of physical activity and cognition. However, I would like to present it with less expectation of it being a research project”. Educators 2–3 (COG): “We would not like to see the program become part of our regular practice. It was difficult to motivate some of the children that were involved in the program, they did not want to participate at the times that were planned or convenient for our program and they children that had not signed up at times were more interested than the others.” Nevertheless, all educators agreed that the program is aligned with the Early Years Learning Framework [30]. Educator 1 (COG): “I think many of the concepts with the story are those within the Framework”.

## 4. Discussion

This study explored the perceptions of early childhood educators and parents of preschool children regarding programs that combine movements with cognitive tasks. For this purpose, firstly an online survey was created and sent out to participants (i.e., early childhood educators’ and parents) in the community, consisting of close- and open-ended questions (Phase 1). Secondly, a program with cognitively engaging physical activity was designed and applied as home-based activities to engage parents and their children, and as school-based activities for preschool centres. Feedback on the program evaluation was received by parents and early childhood educators (Phase 2).

Results from both phases showed that parents of preschool children and early childhood educators have generally consistent opinions regarding the benefits of applying cognitive combined with motor tasks in the home and preschool environment. In general, implementing such programs was positively perceived by parents and early childhood educators, highlighting their potential benefits on children’s learning outcomes, enjoyment and their alignment with the Early Years Learning Framework, the guiding curriculum for Australian preschool children [30]. Enjoyment, linked to inherent motivation can positively enhances students’ academic outcomes [37]. The need for support emerged as a key theme, providing parents and educators with resources, e.g., hands-on activities, songs, extra videos. Regarding program implementation, the delivery dose (i.e., twice per week) and time commitment were appropriate. Positive motivational factors for the children and the teachers included increased confidence and interest, whereas negative factors included different student learning styles, repetition, and class programming.

In particular, Phase 1 was based on previous studies conducting formative research to explore educators’ perceptions on physical activity intervention programs for preschool children [38] as well as their perspectives on technology use [39]. It showed that both parents and educators have a preference of short online programs with duration between 10–30 min, and to be contacted weekly via email. Participants also noted the potential benefits from engaging children to spend quality time on video-based activities promoting their cognitive and physical development, instead of traditional videos or sedentary passive video games. However, time and weather conditions, as well as space limitations emerged as potential barriers. Few participants also mentioned the need for training and special equipment.

Phase 2 focused on the evaluation of the program implementation. Overall, the program was perceived as a positive experience by parents, early childhood educators, and children, with the combination of physical and cognitive tasks being considered as an innovative and interesting component. Children enjoyed participating. Additionally, potential learning benefits emerged for children, especially in the area of counting skills. 

An important finding from Phase 2 was that parents and educators saw the value of the program and were eager to continue its activities beyond its research scope, by adding challenges and making modifications as needed. In order for active learning to be successful, teachers need to implement the lessons as intended, regularly, and accurately. A recent review on active learning (e.g., physical activity breaks or physically active lessons) in primary schools showed that adherence to protocols is quite low, while approximately 50% of the programs are currently researcher led, minimising opportunities for real-world applications [36]. 

Especially when targeting physical activity outcomes, it is well known that preschool children do not meet the physical activity guidelines, whereas opportunities for physically active play led by the teacher (both outdoors and indoors) are limited [40]. Intervention programs focusing on managing and preventing weight loss and/or obesity in preschool children involving physical activity and healthy eating have found mixed effects (12/32 were efficacious [10]). For example, a training program for early childhood educators to promote physical activity participation in preschools did not find any changes on children’s number of steps [41]. Similarly, a review of reviews on school-based physical activity interventions in children and adolescents aged 6–18 years found that 47–65% of trials were effective [42]. Nevertheless, the current study did not include any quantitative measures of physical activity outcomes, thus it is not possible to make such inferences.

Promoting physical activity within the preschool day is essential as it has been found to be positively related to preschool children’s executive function skills, particularly those with low aerobic fitness [43]. In an 8 week intervention program, educators received one hour of online training and were given a book with activities and pictures from recommended literacy books and a CD with music, to implement cognitively engaging physical activity once per day [44]. It was found that preschool children’s attention scores were improved compared to the sedentary control group, following the usual practice. 

It is imperative to understand and correspond to the needs of early childhood educators to deliver and implement successful physical activity programs. To this vein, we need to consider some barriers and suggestions for improvements reported by both parents and educators, arising from Phase 2: Programs need to be flexible to fit educators’ needs while delivery needs to include adjustments suitable for online environments (i.e., larger book size, neutral background, high quality of sound required). Educators also noted that the current program implemented was more suitable for a smaller rather than a larger group of children. Easy access to materials is also important, since the book was easier to use compared to connecting to online resources. Lastly, programs need to include different variations of activities and complexity levels to keep children motivated and engaged.

As evidenced in both phases (Phase 1: “We don’t like to use screens in our centre”) along with the preference of educators in Phase 2 for the book over the videos, early childhood educators reported they were hesitant or reluctant in using screens for educational purposes. This is in consistency with current research reporting that educators have mixed feelings about technology use and its potential benefits in early childhood education [39]. Future research should identify how the possible benefits of technology use can outweigh its harms or associated risks, whereas substantial and systematic effort is required for altering policies to address parents’ and educators’ needs. This is pertinent in a period where a gradual resumption of face-to-face interactions in school and learning spaces occurs.

For instance, educational programs in digital environments that include movements, are considered a healthier way to combine recreational and educational screen time [45,46], taking into account that one in three Australian preschool-aged children may own their own tablet and smartphone, spending up to 26 h per week in front of screens [47]. A recent study found that young children (pre-K and first grade) experienced higher levels of enjoyment when videos with physical activity included higher levels of sensorimotor experiences, in comparisons with videos offering less movement integration [48]. In contrast, teachers reported their preference over calming and less active videos. Nonetheless, this was not the case for our program delivered to preschool children.

Of note, the current study was conducted during the COVID-19 pandemic, a period in which using screens to teach children has become the norm. Even though the necessity and dependency of technology use has been highlighted, it is also possible that educators, parents and children experienced “screen fatigue”, preferring more physical means of teaching. Having the flexibility to perform this program at home or school environment is another strength of this study, considering that many Australian families withdrew their children from ECEC services in response to COVID-19 pandemic [49]. In 2020, 334,800 children 4–5 years were enrolled in a preschool program, whereas 1.3 M children 0–5 years were eligible to receive a child care subsidy to attend an approved ECEC [49].

Overall, this study showed that combing motor and cognitive tasks was positively perceived by early childhood educators and parents of preschool children, and is feasible to become regular part of daily practices. The suggested approach may include small modifications or alterations of current approaches and strategies used by educators. However, it can offer combined physical and cognitive gains to children. In this study, technology was used as an interactive tool to enhance movement and not to replace movement with screen time. Future research could objectively measure if these programs can reduce screen time and increase children’s physical activity levels using accelerometers.

The contribution of this study to the field includes its innovative design and methodology, based on current theoretical frameworks (e.g., COM-B system and movement integration typology [25,35,36] along with the very limited existing evidence on parents’ and early childhood educators’ perceptions on active learning. Limitations also need to be acknowledged: this study included a relatively small sample size. Due to the survey being conducted online and circulated mainly via social media or emails in Phase 1, we are not able to calculate the actual response rate. Additionally, despite the online survey being anonymous (Phase 1), specific feedback on the program components developed was requested via email (Phase 2). Nevertheless, both parents and early childhood educators reported also negative experiences and comments, as suggestions on how to further improve the program. 

## 5. Conclusions

Children can benefit from the potential positive effects of physical activity on cognition, metacognition, academic achievement and mental health [50,51,52]. Combining motor and cognitive tasks could be a way to unite educational and recreational screen time, promote quality time in young children both at home and in the preschool environment, replacing sedentary time with a healthier alternative arising from the physical activity benefits. This is particularly important considering the reach and impact that early childhood education programs can have in young children. 

## Figures and Tables

**Table 1:** Timeline of study components per phase.

Timeline	
Phase 1	November 2020–April 2021
Phase 2	
Home-based program	January–March 2021
	2 weeks of program delivery: variations to complex activities
School-based program	April–June 2021
	3 weeks of program delivery: variations to complex activities

**Table 2:** Examples of activities per intervention group.

Intervention Groups	Activities	Counting	Simple	Complex
Cognitively engaging physical activity	Gross motor movements to overcome obstacles	Animals Simple: 1–20 Complex: Backwards	e.g., Squat jump for big fish and side walking for small fish 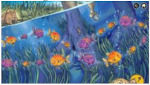	e.g., Opposite actions and different instructions (squat jump—orange fish)
Cognition	Cognitive activities to overcome obstacles without movements	Animals Simple: 1–20 Complex: Backwards	e.g., Say “hiss” when you see a snake and “ribbit” when you see a frog 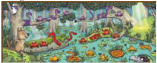	e.g., Say “hiss” when you see a frog and “ribbit” when you see a snake

**Table 3:** Characteristics of study sample.

Characteristics	Parents (*n* = 17)	Early Childhood Educators (*n* = 48)	Total (*n* = 65)
Female participants, n (%)	15 (88.2%)	41 (93.2%)	56
Age			
18–24 years	0	1	1
25–34 years	4	14	18
35–44 years	11	12	23
45–54 years	1	12	13
55–64 years	1	5	6
Language spoken at home			
English	17 (100%)	38 (86.4%)	55 (84.6)
Greek		3	
Polish		1	
Nepali		1	
Cantonese/Chinese		2	
Education			
Year 10 or equivalent (e.g., School Certificate)	0	1	1
Year 12 or equivalent (e.g., Higher School Certificate)	1	1	2
Trade/apprenticeship/certificate (e.g., hairdresser, chef, plumber)	1	3	4
Diploma (e.g., Business/Accounting)	2	11	13
University degree	4	18	22
Post-graduate qualification (e.g., Graduate Diploma, Masters, PhD)	9	10	19
Occupation			
Employed full-time	4	26	30
Employed part-time	12	13	25
Unemployed	1	1	2
Maternity leave		1	
Casual		2	
Self-employed		1	
Years of experience (for early childhood educators)			
0–5 years		13	
6–10 years		2	
11–15 years		11	
16–20 years		5	
20+ years		7	
Location			
ACT		3	3
NSW	13	23	36
QLD		3	3
SA	2	2	4
VIC		2	2
WA	1	8	9
Centrelink card			
No	13	39	52
Yes	3	5	8

**Table 4:** Participant responses regarding preferred duration of program delivery.

Program Duration	Early Childhood Educators	Parents
10–15 min	11	5
15–30 min	14	6
30–45 min	8	2
45–60 min	3	1

**Table 5:** Participant responses regarding potential benefits.

	Early Childhood Educators	Parents
Physical activity levels	29	10
Engagement during learning	24	8
Cognitive function	28	8
Mood	23	8
Sleep	19	9
Learning outcomes	22	8
Quality time with children	22	7

## Data Availability

The data presented in this study are available on request from the corresponding author. The data are not publicly available.

## Appendix A. Phase 1: Online Survey

Program delivery, duration, and frequency

1. If we offered you an intervention program, would you prefer to be delivered in:
  ■ A hard copy (e.g., book)
  ■ electronic version (e.g., videos)
2. We would like to establish an ongoing communication with you during the implementation of an intervention. What means would you consider as the easiest to get in touch with you?
  ■ Email
  ■ Group chat
  ■ Text message
  ■ Mobile app
  ■ Facebook
  ■ hardcopy logbook/diary
3. What do you think would be the ideal duration of a training session?
  ■ 10–15 min
  ■ 15–30 min
  ■ 30–45 min
  ■ 45–60 min
4. If we were interested in collecting information on children’s daily behaviours (sleep time, screen time, physical activity, engagement and enjoyment during the program), how often do you think you would be able to provide this information for 4–6 weeks?
  ■ Daily
  ■ 1 a week
  ■ 2–3 times per week
  ■ 1 a fortnight

Benefits

5. If we offered you a video-based intervention (showing physical activities combined with learning tasks) to use it during screen time. Would you consider it as an engaging way to spend quality time with your children as an alternative instead of traditional videos or video games?
  ■ Yes
  ■ No

Comments: _______________________________________________

6. Are there any benefits that can arise related to physical activities combined with learning tasks (e.g., combined physical and cognitive benefits) or at day care/preschools (for early childhood educators)?
  ■ Increased physical activity levels per day
  ■ Engagement during learning
  ■ Better sleep
  ■ Quality time with children
  ■ Better learning
  ■ Improved cognitive function (e.g., concentration, attention)
  ■ Better mood
  ■ Other
7. Do you consider interventions that combine physical activities with learning tasks as an effective way to reduce sitting time in young children?
  ■ Yes
  ■ No

Comments: _______________________________________________

8. Do you think physical activity can improve children’s self-regulation?
  ■ Yes
  ■ No

Comments: _______________________________________________

Barriers

9. Are there any barriers in implementation that can be related to physical activities combined with learning tasks at home (for parents) or at day care/preschools (for early childhood educators)?
  ■ Time restrictions
  ■ Need for special equipment
  ■ Space restrictions
  ■ Need for training
  ■ Other

## Appendix B. Phase 2: Post-Program Evaluation Feedback Questionnaire

For parents

1. What is your overall experience of the Quincey Quokka’s research program?
2. Was there anything that you really liked about the program?
3. Is there anything that you suggest we should change?
4. Have you noticed an impact on your child? (e.g., motivation, enthusiasm and/or enjoyment during reading sessions, improved counting, improved self-regulation)
5. Did your child enjoy the program? Do you know why (e.g., activities or story)?
6. Do you believe that you could keep using some of the activities at home? (Yes, No and why)?

For Educators

1. What is your overall experience of the Quincey Quokka’s research program?
2. Was there anything that you really liked about the program?
3. Is there anything that you suggest we should change?
4. Have you noticed an impact on your children? (e.g., motivation, enthusiasm and/or enjoyment during reading sessions, improved counting, improved self-regulation)
5. Did children enjoy the program? Do you know why (e.g., activities or story)?
6. What were/are some of the barriers and/or enablers that have arisen to delivering the program in your school?
7. What have been the benefits of integrating physical activity with activities enhancing cognition during the preschool day?
8. Do you think the Quincey Quokka’s Research Program is aligned with the Early Years Framework?
9. Do you believe that Quincey Quokka’s Research Program could become part of your regular practice? (Yes, No and why)?

## Appendix C

**Table A1:** Summary of Thematic Analyses from Phases 1 and 2.

Theme	Sub-Theme	Content	Quotes
Design	Teacher-driven	- Expanded activities besides the video/book	- It was a great experience and inspired me to use similar activities in daily reading/play to help my child to develop his math and other cognitive ability. (P2- H/P1; CPA) - Yes, as we have seen the benefits in the short time and the children have been asking for us to still read the book and do the movements even in other books with the same animals. (P2-Sch/T2; CPA)
	Research-teacher collaboration	- Adjustments were made as needed: explanation of instructions, model activities, be a large group activity for preschool centre. - Communication with research team	- It was a positive experience, however, my son needed encouragement to begin each time. His engagement improved each time, though. I found that I had to explain the instructions to him sometime as he was listening closely enough, and I would need to do the activities with him at times, to either model for him, or to keep him engaged. (P2-H/P2; CPA) - Our experience was good however it was hard to follow along with the book and instructions in the video link so we found it easier to read the children the book ourselves and then do the movement instruction as we read. The video story was hard to hear for a larger group of children. (P2-Sch/T2; CPA) - Engaging in professional discussions with the team of researchers. The opportunity to contribute to building a local research base of knowledge. (P2-Sch/T2; CPA) - During our planned group times many of the children were keen to participate. At times the children who were involved in the program did not want to do it. We heard comments such as “No thank you I don’t need to see that”. For this reason, we decided to make the Quokka Time story a larger group activity. This way it captured all of the children that were participating and they did not feel like they were taken out of the preschool program to do something that they were not ready to do at a particular time. (P2-Sch/T2; COG)
	Research-driven (video)	- No need for interaction with “strangers” - Independent implementation	- Absolutely, a beneficial way to combine quality time and engagement with my children whilst also exploring physical activity, education and technology use. I must admit, I am a mum that can struggle with continually finding meaningful ways (P1) - This was a great approach for my very shy child who lacks confidence interacting and communicating with new people. The fact that she didn’t really need to engage with a ‘stranger’ was a real advantage for us. The iPad testing is perhaps the only means by which our participation was possible—if this testing required interaction with a new and different person, we probably would not have got past the first question. The video was a simple delivery method allowing us all to engage with the activity. Having the flexibility to watch the video at any time was a distinct pro for us. Our experience with the video and being led through activities meant that we were easily able to understand and engage with other new pages/activities in the book. Discovering what else was in the book was a real treat for us all. (P2-H/P1; COG)
Strategy	Integrated	- Relevance and integration with movement	- I like how it embeds math in activities and makes it interesting to children. (P2-H/P2; CPA) - They especially liked the activity that make the birds sound, all the activities were fun and easy to follow. (P2-H/P2; COG) - We loved the movements with the book and the children were able to pick up the movements quickly when we were reading. (P2-Sch/T2; CPA)
	Expanding opportunities	- Alternative ways of reading - Adding Challenges	- I liked that it incorporated physical activity into reading. This is something we don’t usually do as reading is usually a calming activity before they nap or sleep at night. This would encourage me to read at other times through the day (P2-H/P1; CPA) - We were able to relate this notion to concepts that were familiar to them, for example challenges they see on TV or video games, where they have to successful in the challenge before they can get to the next level (P2-Sch/T2; COG) - Whilst we have not revisited the video, the book does often appear in our daily reading time together. The activities are enjoyed by the kids and the ability for parents to adjust the complexity of some activities to suit the child is great—we can throw a little challenge out there and see how it is received and keep the interest and engagement continuing. (P2-H/P1; COG)
Support	Resources	- Use of iPad - Physical copy of book	- iPad testing—The concept of playing games was actually something for her to look forward to and enjoy. (P1) - The book itself was interesting and interactive to older children in the group. (P2-Sch/T1; COG) - LOVED receiving her very own copy of the book. Reading along at home and discovering new and different activities as well as familiar ones was a real source of enjoyment and excitement for my kids. Also, with their very own book, they quite enjoyed taking on the narrator’s role from the video, leading the activities and requiring me to respond! (P2-H/P1; COG) - As a parent of a very shy and reserved child, the ‘testing’ on iPads was great. I was able to encourage her participation with a promise of playing games on the iPad!! (P2-H/P1; COG)
	Lack of resources	- Songs - Hands-on activities - Multiple copies of resources - More videos	- If it contained songs and engaging content. (P1) - If Quincey was to become a tool in my daily work, I would think focussing on one section at a time and providing additional hands-on activities to embed the concepts in children’s learning. A bigger format book would also be useful, along with additional props (such as shape stepping stones) for more experiential learning. (P2; Sch/T1; CPA) - My suggestion is to provide more videos with different activities (P2-H/P2; COG) - Needed the same resource for approximately 3 or 4 small groups (P2-Sch/T1; COG)
	Researchers	- Provide ideas and support when needed	- Research staff was wonderful—incredibly understanding and patient with my child who at times just wasn’t quite sure. Her communication was professional, timely and helped to keep us on track with our commitments to the program. (P2-H/P1; COG) - Enablers were the support of the research staff providing extension ideas and making herself available if needed. (P2-Sch/T1; CPA)
	Extra Training	-Extra training for educators	- Also some extra training for educators would be useful, so that we are better equipped to implement the program. We felt that the training was quite limited. (P2-Sch/T2; COG)
Delivery	Type	- Preference for book and not screen - Preference for outdoor vs. indoor	- I don’t like to use screens around the children in our centre. They have enough of that out of hours. (P1) - This would limit us to being inside and I have found screen time learning does not hold all children’s attention (P1) - Watching and engaging with videos in an early childhood setting doesn’t provide a way to connect with the children whilst doing the activity as they are mostly focused on the screen. (P1) - We don’t use screens with children (P1) - I like that the videos give different options using the same book, but I do prefer doing them while reading the book, and not through the video (P2-H/P3; CPA)
		- Preference for video	- The video was also an appropriate length, it didn’t drag on and we could all maintain our attention. The book’s narrator came across warm and friendly, encouraging our kids to participate. (P2-H/P1; COG)
		- Combination of video and book	- Whilst the pages led through the video were good, being able to later repeat these activities on our own with our very own book was a real positive and something my kids loved (even the one that didn’t participate). (P2-H/P1; COG) - If we have a copy of the book, show it to him directly and give him enough time to think and react, it probably will work better. (P2-H/P2; CPA)
	Technical difficulties	- Position and size of book - Sound - Quality of video	- If possible, it would’ve helped if the book was closer to the screen so the child could see and engage with the story and pictures more.” (P2-H/P1; CPA) - To begin with, we struggled to see some of the detail (colours, pictures) on the pages of the book, in the video. At first, I felt that it may be handy to have a copy of the book with us from the offset, to read along with the video… although, perhaps that may have been a distraction. (P2-H/P1; COG) - The book was in too small a format for large groups of children. The sound quality of the video was not clear (possibly our set up?) and the book images were also small (even on our large screen). It was hard to maintain the children’s motivation to participation due to the repetition, even when we mixed it up with different delivery methods. (P2-Sch/T1; CPA) - The book was too busy and detailed for a large group to focus and engage. The story worked much better with only a small group. The concept of changing the names of animals was difficult for some of the children who are only just beginning to learn English. (P2-Sch/T1; COG) - If we could recommend anything we would suggest making the book larger as we are used to using large books with children at group time. (P2-Sch/T2; COG) - In addition, the picture from the book is not clear/big enough to see in the video, especially in colourful background. Sometimes there is not enough time for him to think and react. Most of the time my son copies my movement or instructor’s movement instead of thinking and reacting by himself. (P2-H/P2; CPA)
	Dose	- Time	- The time commitment was reasonable, the activities were enjoyable and the length of the videos and expectations around record keeping was achievable. (P2-H/P1; CPA)
	Fidelity	- Consistency on delivery	- Also, ensure every educator is delivering the program as intended. (P2-Sch/T1; COG)
Motivation	Positive	- Enjoyment - Confidence - Interest	- We had fun participating in the program. The activities are fun and useful, and my 3 kids are enjoying doing them together. (P2-H/P3; CPA) - I can’t say I have noticed any obvious or overt impacts. My child has always enjoyed reading books and will sit still and contently listen as I read and loves following along by looking at the pictures. I don’t feel her enthusiasm for or enjoyment of reading has changed, but it would be hard to get much keener than she is! If anything, she has possibly gained more confidence and accuracy with her counting. For a child who is reluctant to count or provide answers for fear of getting it wrong, she does seem to be more willing to count, be heard and to answer questions around numbers—the fear of getting the answer wrong is still strong but the confidence to engage is there. This is a positive. (P2-H/P1; COG) - I enjoyed the challenge of keeping the children and myself motivated. (P2-Sch/T1; CPA) - We saw an improvement in our children’s interest and enthusiasm (P2-Sch/T2; CPA) - We did like that the program involved a series of Quests. We found that this concept was particularly motivating for the children and the boys in particular. (P2-Sch/T2; COG) - We noticed that during your assessment visits many children were keen to see what it was all about. The iPad assessment games appeared to be very engaging and this attracted other children. (P2-Sch/T3; COG)
	No changes		- I didn’t notice too much difference on motivation/enjoyment. He always has great interest and enjoyment in reading and that stays the same. (P2-H/P2; CPA) - I haven’t noticed an impact on my kids yet. (P2-H/P2; COG) - With regards improved counting, enjoyment and self-regulation, we would say that we did not see improvement. (P2-Sch/T2; COG)
	Negative	- Lack of variety - Learning style - Repetition - Task difficulty - Class programming	- He enjoyed it very much for the first 2 or 3 times and engaged from the beginning to the end with great interest. After that he lost his interest gradually and I think it is because there is limited variation in activities and the story stays the same. He refused to watch the video again for the last few times. (P2-H/P2; CPA) - I wondered often on the learning intention behind the program and if the children were listening and interpreting the instructions [due to problems with sound] or just mimicking the presenter’s actions on the screen. I also needed to work hard to maintain my enthusiasm when the children became reluctant. (P2-Sch/T1; CPA) - Although many children were motivated to join in the activity at group time and we attempted to engage the children as a small and larger group, often we found that the children were not engaged by the activities. They were very repetitive and at times difficulty to engage the children’s attention for long. (P2-Sch/T2; COG) - Perhaps more variation in the video’s (more than just two versions) would have maintained a greater enthusiasm for engagement with my kids. (P2-H/P1; COG)
Benefits	Academic, Cognitive, and Motor	- Counting - Reading - Comprehension - Reaction Time - Holistic development - Movement skills - Alignment with Early Years Learning Framework	- His counting has improved a bit, especially trying to count backward, as that is something I had not focused on before. (P1-H/P1; CPA) - When I asked him what the book was about after a few readings he was able to tell me more than I anticipated. (P2-H/P1; CPA) - His counting improved a little bit (can count above 13 until 18 and can count backwards).(S2-H/P2; CPA) - For me the Quincey Quokka story book bought together many of the focus learning opportunities we provide to children throughout our day at preschool. (P2-Sch/T1; CPA) - I feel my son masters the concept better with physical activities and it also trains his reaction (like doing the opposite movement, remembering the order, etc.) (P2-H/P2; CPA) - We saw an improvement in counting throughout the program and still now. The children are also asking for the book to be read so they can do the movements. (P2-Sch/T2; CPA) - As we offer an environment rich in these kinds of intentional teaching, focused learning I could say that the Quincey book alone contributed to noticeable shifts in these areas. (P2-Sch/T1; CPA) - It sits within Outcome 3: physical activity, emotional regulation areas, as well as Outcome 4 learning dispositions and Outcome 5 communication learning. (P2-Sch/T1; CPA) - Yes we do believe that the Quincey Quokka research program is aligned with the EYLF, especially when it is utilised in small groups (P2-Sch/T2; COG)
Barriers		- Use of accelerometers - Staff rotation - Child attendance/Illnesses	- The children did seem to enjoy the program. Some tended not to want to involve when accelerometers were added. There tends at this age to be a bit of a domino effect when one child decides not to participate. (P2-Sch/T1; COG) - Time management, not all staff on board with the program and not all children engaging each day. (P2-Sch/T2; CPA) - The barriers include the fact that during the program implementation several of the children were absent due to illness. When children are absent for one or more weeks, then it is difficult to assess whether the program is of benefit as there is limited interaction and ongoing participation. (P2-Sch/T2; COG)
Opportunities	Parents	- Transfer of learning - Disseminating research finding	- It would also be a useful tool to share with families to transfer this learning to home. I think this book is best used in a one on one or small group which would enable the adult to support the child where they were currently at. (P2-Sch/T1; CPA) - Overall, we did enjoy the experience and particularly learning a bit more about your research and the outcomes. It will be valuable for us to receive an update of the findings of your research as we think it will be valuable to share this information with families, especially of those who participated in the program. (P2-Sch/T2; CPA)
	Childcare centres	- Enriched childcare programming	- As a mum who wants to provide more of this type of opportunity for my children, I feel the childcare centre attend lack enough programming, particularly in this area. I would love to see this concept/program introduced in my child’s centre. (P1)

Note: P = phase; T = teacher; P = parent; Sch = school-based program; H = home-based program; CPA = cognitively engaging PA group; COG = cognition group.

## References

[1] Brown T.T., Jernigan T.L. (2012). Brain development during the preschool years. Neuropsychol. Rev..

[2] Carson V., Lee E.Y., Hewitt L., Jennings C., Hunter S., Kuzik N., Stearns J.A., Unrau S.P., Poitras V.J., Gray C. (2017). Systematic review of the relationships between physical activity and health indicators in the early years (0–4 years). BMC Public Health.

[3] World Health Organisation (2019). Guidelines on Physical Activity, Sedentary Behaviour, and Sleep for Children under 5 Years of Age. https://www.who.int/news/item/24-04-2019-to-grow-up-healthy-children-need-to-sit-less-and-play-more.

[4] Hills A.P., Dengel D.R., Lubans D.R. (2015). Supporting public health priorities: Recommendations for physical education and physical activity promotion in schools. Prog. Cardiovasc. Dis..

[5] Coleman B., Dyment J.E. (2013). Factors that limit and enable preschool-aged children’s physical activity on child care centre playgrounds. J. Early Child. Res..

[6] Ellis Y.G., Cliff D.P., Okely A.D. (2018). Childcare educators’ perceptions of and solutions to reducing sitting time in young children. Early Child. Educ. J..

[7] Ernst J., Tornabene L. (2012). Preservice early childhood educators’ perceptions of outdoor settings as learning environments. Environ. Educ. Res..

[8] Macdonald K., Milne N., Pope R., Orr R. (2021). Factors influencing the provision of classroom-based physical activity to students in the early years of primary school: A survey of educators. Early Child. Educ. J..

[9] Martínez-Bello V.E., del Mar Bernabé-Villodre M., Lahuerta-Contell S., Vega-Perona H., Giménez-Calvo M. (2021). Pedagogical knowledge of structured movement sessions in the early education curriculum: Perceptions of teachers and student teachers. Early Child. Educ. J..

[10] Akamoglu Y., Ostrosky M.M., Cheung W.C., Yang H.W., Favazza P., Stalega M., Aronson-Ensign K. (2019). Move together, communicate together: Supporting preschoolers’ communication skills through physical activities. Early Child. Educ. J..

[11] Michael R.D., Webster C.A., Egan C.A., Nilges L., Brian A., Johnson R., Carson R.L. (2019). Facilitators and barriers to movement integration in elementary classrooms: A systematic review. Res. Q. Exerc. Sport..

[12] Ling J., Robbins L.B., Wen F. (2016). Interventions to prevent and manage overweight or obesity in preschool children: A systematic review. Int. J. Nurs. Stud..

[13] Fuller A.B., Byrne R.A., Golley R.K., Trost S.G. (2019). Supporting healthy lifestyle of facilitators and barriers. BMC Public Health.

[14] Bedard C., St John L., Bremer L., Graham J.D., Cairney J. (2019). A systematic review and meta-analysis on the effects of physically active classrooms on educational and enjoyment outcomes in school age children. PLoS ONE.

[15] Daly-Smith A.J., Zwolinsky S., McKenna J., Tomporowski P.D., Defeyter M.A., Manly A. (2018). Systematic review of acute physically active learning and classroom movement breaks on children’s physical activity, cognition, academic performance and classroom beehavior: Understanding critical design features. BMJ Open Sport Exerc. Med..

[16] Norris E., van Steen T., Direito A., Stamatakis E. (2019). Physically active lessons in schools and their impact on physical activity, educational, health and cognition outcomes: A systematic review and meta-analysis. Br. J. Sports Med..

[17] Watson A., Timperio A., Brown H., Best K., Hesketh K.D. (2017). Effect of a classroom-based physical activity interventions on academic and physical activity outcomes: A systematic review and meta-analysis. Int. J. Behav. Nutr. Phys. Act..

[18] Mavilidi M.F., Okely A.D., Chandler P., Cliff D.P., Paas F. (2015). Effects of integrated physical exercises and gestures on preschool children’s foreign language vocabulary. Educ. Psychol. Rev..

[19] Mavilidi M.F., Okely A.D., Chandler P., Paas F. (2016). Infusing physical activities into the classroom: Effects on preschool children’s geography learning. Mind Brain Educ..

[20] Mavilidi M.D., Okely A.D., Chandler P., Paas F. (2017). Effects on integrating physical activities into a science lesson on preschool children’s learning and enjoyment. Appl. Cogn. Psychol..

[21] Mavilidi M.F., Okely A., Chandler P., Domazet S.L., Paas F. (2018). Immediate and delayed effects of integrating physical activity into preschool children’s learning of numeracy skills. J. Exp. Child. Psychol..

[22] Mavilidi M.F., Ruitter M., Schmidt M., Okely A.D., Loyens S., Chandler P., Paas F. (2018). A narrative review of school-based physical activity for enhancing cognition and learning: The importance of relevancy and integration. Front. Psychol..

[23] Vazou S., Pesce C., Lakes K., Smiley-Oyen A. (2019). More than one road leads to Rome: A narrative review and meta-analysis of physical activity intervention effects on cognition in youth. Int. J. Sport Exerc. Psychol..

[24] Diamond A., Lee K. (2011). Interventions shown to aid executive function development in children 4 to 12 years old. Science.

[25] Schmidt M., Mavilidi M.F., Singh A., Englert C. (2020). Combining physical and cognitive training to improve kindergarten children’s executive functions: A cluster randomized controlled trial. Contemp. Educ. Psychol..

[26] Melhuish E.C. (2011). Preschool matters. Science.

[27] Turner L., Calvert H.G., Carlson J.A. (2019). Supporting teachers’ implementation of classroom-based physical activity. Transl. J. Am. Coll. Sports Med..

[28] Vazou S. (2019). From “Sit still and listen” to “Get up and move”, the leap may be one of educational paradigms but no longer one of faith. Transl. J. Am. Coll. Sports Med..

[29] Webster C.A., Zarrett N., Cook B.S., Egan C., Nesbitt D., Weaver R.G. (2017). Movement integration in elementary classrooms: Teacher perceptions and implication for program planning. Eval. Program Plann..

[30] Australian Government Department of Education and Training (2009). Belonging, Being, & Becoming: The Early Years Learning Framework for Australia. Canberra, ACT: Commonwealth of Australia. https://www.acecqa.gov.au/sites/default/files/2018-02/belonging_being_and_becoming_the_early_years_learning_framework_for_australia.pdf.

[31] Pesce C., Vazou S., Benzing V., Álvarez-Bueno C., Anzeneder S., Mavilidi M.F., Leone L., Schmidt M. (2021). Effects of chronic physical activity on cognition across the lifespan: A systematic meta-review of randomized controlled trials and realist synthesis of contextualized mechanisms. Int. Rev. Sport Exerc. Psychol..

[32] Howard S.J., Powell T., Vasseleu E., Johnstone S., Melhuish E. (2017). Enhancing preschoolers’ executive functions through embedding cognitive activities in shared book reading. Educ. Psychol. Rev..

[33] Riley N., Mavilidi M.F., Kennedy S.G., Morgan S.G., Lubans D.R. (2021). Dissemination of “Thinking while moving in maths”: Implementation barriers and facilitators. Transl. J. Am. Coll. Sports Med..

[34] Braun V., Clarke V. (2006). Using thematic analysis in psychology. Qual. Res. Psychol..

[35] Mitchie S., Van Stralen M.M., West R. (2011). The behaviour change wheel: A new method for characterising and designing behaviour change interventions. Implement. Sci..

[36] Vazou S., Webster C.A., Stewart G., Candal P., Egan C.A., Pennell A., Russ L.B. (2020). A systematic review and qualitative synthesis resulting in a typology of elementary classroom movement integration interventions. Sports Med. Int. Open..

[37] Deci E.L., Ryan R.M. (1985). Intrinsic Motivation and Self-Determinism in Human Behavior.

[38] Foulkes J.D., Foweather L., Fairclough S.J. (2020). “I wasn’t sure what it meant to be honest”—Fomative research towards a physical literacy intervention for preschoolers. Children.

[39] Zabatiero J., Straker L., Mantilla A., Edwards S., Danby S. (2018). Young children and digital technology: Australian early childhood education and care sector adults’ perspectives. Australas. J. Early Child..

[40] Tandon P.S., Saelens B.E., Christakis D.A. (2015). Active play opportunities at child care. Pediatrics.

[41] Mavilidi M.F., Rigoutsos S., Venetsanou F. (2021). Training early childhood educators to promote children’s physical activity. Early Child. Educ. J..

[42] Kriemler S., Meyer U., Martin E., van Sluijs E.M., Andersen L.B., Martin B.W. (2011). Effect of school-based interventions on physical activity and fitness in children and adolescents: A review of reviews and systematic update. Br. J. Sports Med..

[43] Becker D.R., Abi Nader P. (2021). Run fast and sit still: Connections among aerobic fitness, physical activity, and sedentary time with executive function during pre-kindergarten. Early Child. Res. Q..

[44] Vazou S., Mavilidi M.F. (2021). Cognitvely engaging physical activity for targeting motor, cognitive, social, and emotional skills in the preschool classroom: The Move for Thought preK-K program. Front. Psychol..

[45] Straker L.M., Zabatiero J., Danby S., Thorpe K., Edwards S. (2018). Conflicting guidelines on young children’s screen time and use of digital technology to create policy and practice dilemmas. J. Pediatr..

[46] Straker L.M., Howie E.K. (2016). Young children and screen time: It is time to consider healthy bodies as well as healthy minds. J. Dev. Behav. Pediatr..

[47] Rhodes A. (2017). Screen Time and KIDS: What’s Happening in Our Homes?. www.childhealthpoll.org.au/wp-content/uploads/2017/06/ACHP-Poll7_Detailed-Report-June21.pdf.

[48] Strapp A.C., Prior L.F., Smith C.H. (2021). Moving across Mississippi: Young children’s enjoyment and teachers’ perceptions of standards-based physical activity videos. Teach. Teach. Educ..

[49] Australian Government, Australian Institute of Health and Welfare (2021). Childcare and Early Childhood Education. https://www.aihw.gov.au/reports/australias-welfare/childcare-and-early-childhood-education.

[50] Álvarez-Bueno C., Pesce C., Cavero-Redondo I., Sánchez-Lopez M., Martínez-Hortelano J.A., Martinez-Vizcaino V. (2017). The effects of physical activity interventions on children’s cognition and metacognition: A systematic review and meta-analysis. J. Am. Acad. Child. Adolesc. Psychiatry.

[51] Álvarez-Bueno C., Pesce C., Cavero-Redondo I., Sánchez-Lopez M., Garrido-Miguel M., Martinez-Vizcaino V. (2017). Academic achievement and physical activity: A meta-analysis. Pediatrics.

[52] Biddle S.J., Asare M. (2011). Physical activity and mental health in children and adolescents: A review of reviews. Br. J. Sports Med..

